# The Effect of Ginger (*Zingiber officinale*) on Platelet Aggregation: A Systematic Literature Review

**DOI:** 10.1371/journal.pone.0141119

**Published:** 2015-10-21

**Authors:** Wolfgang Marx, Daniel McKavanagh, Alexandra L. McCarthy, Robert Bird, Karin Ried, Alexandre Chan, Liz Isenring

**Affiliations:** 1 Health Sciences & Medicine, Bond University, Gold Coast, Queensland, Australia; 2 Division of Cancer Services, Princess Alexandra Hospital, Brisbane, Queensland, Australia; 3 Institute of Biomedical Innovation, Queensland University of Technology, Brisbane, Queensland, Australia; 4 School of Medicine, Griffith University, Queensland, Australia; 5 National Institute of Integrative Medicine, Melbourne, Victoria, Australia; 6 Department of Pharmacy, Faculty of Science, National University of Singapore, Singapore, Singapore; 7 Department of Nutrition & Dietetics, Princess Alexandra Hospital, Queensland, Australia; University of Leuven, BELGIUM

## Abstract

**Background:**

The potential effect of ginger on platelet aggregation is a widely-cited concern both within the published literature and to clinicians; however, there has been no systematic appraisal of the evidence to date.

**Methods:**

Using the PRISMA guidelines, we systematically reviewed the results of clinical and observational trials regarding the effect of ginger on platelet aggregation in adults compared to either placebo or baseline data. Studies included in this review stipulated the independent variable was a ginger preparation or isolated ginger compound, and used measures of platelet aggregation as the primary outcome.

**Results:**

Ten studies were included, comprising eight clinical trials and two observational studies. Of the eight clinical trials, four reported that ginger reduced platelet aggregation, while the remaining four reported no effect. The two observational studies also reported mixed findings.

**Discussion:**

Many of the studies appraised for this review had moderate risks of bias. Methodology varied considerably between studies, notably the timeframe studied, dose of ginger used, and the characteristics of subjects recruited (e.g. healthy vs. patients with chronic diseases).

**Conclusion:**

The evidence that ginger affects platelet aggregation and coagulation is equivocal and further study is needed to definitively address this question.

## Introduction

There is increasing evidence that ginger and its constituents might exert meaningful anti-nausea effects during cancer chemotherapy. Our recent systematic review of the literature found preliminary evidence that supported its use as an adjuvant anti-nausea drug to standard anti-emetics in the chemotherapy setting.[1] Concerns over potential “off target” antiplatelet effects, however, could limit the application of ginger in oncology patients, who frequently experience thrombocytopenia due to myelosuppression.

The ginger rhizome has been used in traditional systems of medicine for centuries and more recently, its potentially medicinal properties have been empirically studied.[2] Current research suggests that the active constituents of ginger, namely the gingerol and shogaol classes of compounds, might exert several beneficial effects including anti-inflammatory, antioxidant, and cholesterol lowering properties.[2] In addition, ginger is a promising treatment for nausea associated with a variety of stimuli including post-operative nausea and vomiting, motion sickness, morning sickness, and chemotherapy-induced nausea and vomiting.[1, 3–5]

While the safety profile of ginger supplementation requires further investigation, previous clinical trials report few side-effects, mostly minor in nature (e.g. mild nausea, heartburn).[1] Of these reported side effects, potentially the most significant is an antiplatelet effect. Two published case-studies reported adverse symptoms and abnormal platelet aggregation that was temporally related to recent ingestion of ginger products.[6, 7] In addition, several animal and *in vitro* studies have reported ginger as well as individual ginger compounds to have an effect on platelet aggregation.[8–10] While this action could be beneficial in vascular diseases, it could potentiate bleeding risk in conditions such as thrombocytopenia or pre-existing platelet dysfunction. This is particularly relevant in the chemotherapy setting, where therapy-induced thrombocytopenia is associated with treatment delays, dose reductions, and bleeding events.[11]

To the authors knowledge, Srivastava et al.[8] were the first group to investigate the effect of ginger on platelet aggregation by using four ginger extracts, produced using different solvents (aqueous, n-hexane, chloroform, and ethyl acetate). They reported that ginger inhibited platelet aggregation using arachidonic acid (AA), epinephrine, adenosine diphosphate (ADP), and collagen as agonists. Others have corroborated this, reporting that certain ginger compounds inhibit *in vitro* platelet aggregation when using a variety of agonists (AA, collagen, platelet activating factor, and thrombin).[12, 13] This reduction in platelet aggregation was most potent when AA was used as the agonist, requiring lower concentrations to cause inhibition when compared to the other agonists.[9, 12]

While few studies investigating the effect of ginger and its compounds on the clotting cascade have been undertaken, a considerable amount of *in vitro* research suggests that ginger compounds interact with AA-derived eicosanoid and thromboxane synthesis.[14–18] The AA cascade can produce the eicosanoids involved in inflammation (i.e. prostaglandin E2) as well as thromboxane, which is amongst the many agonists of platelet aggregation. Numerous studies indicate that ginger extract and particular ginger compounds inhibit products specific to the cyclooxygenase pathway, including a reduction in thromboxane B_2_ (TxB_2_) production,[19] prostaglandin formation (PGF2a, PGE_2_, and PGD_2_),[8, 15] and cyclooxygenase enzyme activity.[16, 18] These same compounds also interact with the lipoxygenase pathway, including reductions in 5-lipoxygenase enzyme activity.[14] Finally, ginger compounds might also inhibit the activity of phospholipase A_2,_ which suggests that ginger exerts its anti-platelet aggregating as well as its potential anti-inflammatory actions through interaction with one of the initial steps in this pathway.[20]

Due to the observed *in vitro* effects of ginger on the AA cascade, excessive bleeding and interactions with platelet therapy during cancer chemotherapy are of clinical concern. While the results of *in vitro* studies are consistent, these results are not always translatable to the complex human system. Clinical and observational data, however, provide a reasonable indication of the potential human response. There is a growing body of clinical and epidemiological literature in this area, although no systematic appraisal of the relevant literature has been undertaken to date. In this paper, we summarise and discuss the findings of clinical and observational studies regarding the effect of ginger, compared to placebo or baseline, on platelet aggregation in multiple participant populations.

## Methodology

### 2.1 Data Sources and Searches

Using the Preferred Reporting Items for Systematic Reviews and Meta-Analyses (PRISMA) guidelines,[21] a systematic search of the literature was conducted using the following databases: MEDLINE, CiNAHL, Embase, and Cochrane Library. The reference lists of retrieved papers were also searched for additional manuscripts. Search terms were not limited by a specific timeframe; rather, all search queries were from the date of the journal’s inception to May 2014. Search terms were broad so as to ensure all relevant manuscripts were captured.

### 2.2 Study Selection

The search terms used were “ginger AND (platelet OR thrombo* OR clot* OR bleed OR “adverse effects” OR “side effects” OR haemorrhage)”. Studies included in this review 1) were written in English 2) stipulated the independent variable was a ginger preparation or isolated ginger compound, and 3) used measures of platelet aggregation as the primary outcome.

### 2.3 Data Extraction and Quality Assessment

Extracted data included: participant demographic (e.g age, gender, reported comorbidities), type of ginger intervention (e.g dosage, timing, form of ginger), study design characteristics (e.g. sample size, risk of bias, type of study, study length), and reported outcomes (e.g measures of platelet aggregation, adverse events, dropout rates).

All clinical studies were individually rated for evidence level by author WM using the National Health and Medical Research Council Hierarchy of Evidence guidelines (IV-I, with I being the strongest level of evidence).[22] They were also independently assessed for bias, by two authors (WM and DM) using the Cochrane Handbook for Systematic Reviews of Interventions checklist.[23] Where insufficient information was included in the manuscript to assess particular forms of bias, further information was sought via correspondence with the study authors. Blinding is unlikely to affect the results of the clinical biomarkers measured in these studies, hence trials that were not blinded were rated in the review as low-risk for detection and performance included bias. In addition, due to the small number of trials in this area, no study was excluded based on its risk of bias.

### 2.4 Data Synthesis and Analysis

A statistically significant (P≤0.05) result was considered evidence of an effect. Relevant study details were retrieved from their respective manuscript using a standardised form. Forest plot and meta-analysis was intended; however, due to the heterogeneity of the studies included in this review, these analyses were found to be unfeasible.

## Results

**Fig 1:**
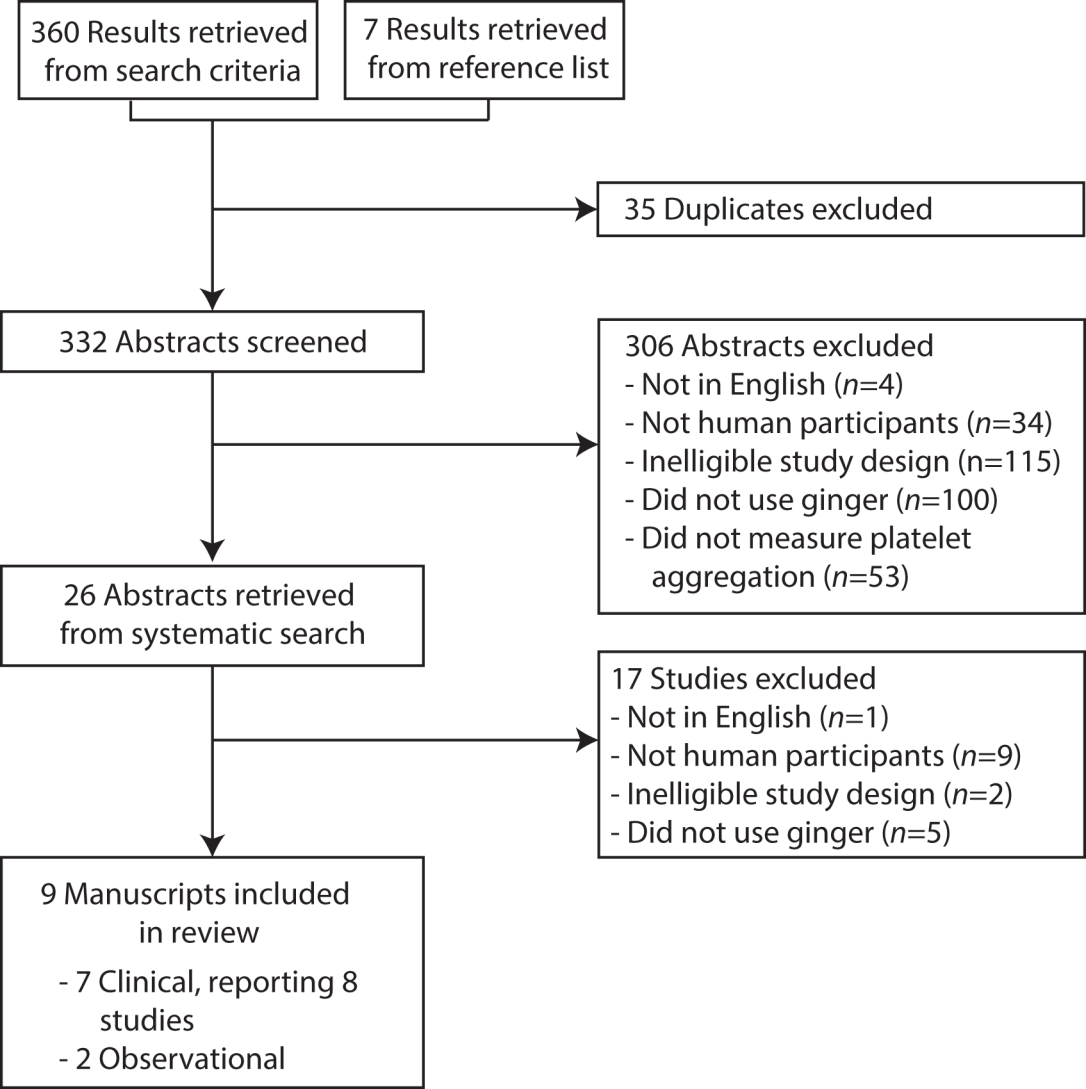
PRISMA study flow diagram.

A total of 367 papers were identified (Fig 1). After assessment of study abstracts and the removal of duplicates 26 abstracts were retrieved for further examination. Seventeen were subsequently excluded, resulting in 9 manuscripts included in the final review.

### 3.1 Clinical Trials

Seven manuscripts reporting the effect of ginger on platelet aggregation in human participants using a clinical trial design were retrieved (Table 1). Of the seven manuscripts, one described two separate trials, resulting in a total of eight clinical trials included in this review.[24]

**Table 1:** Extraction table of reviewed clinical trials.

Author/Date	Study design	Time points	Population	Intervention	Outcome	Results	Country	Level of evidence	Comment
Bordia et al. 1997	Placebo-controlled trial	Total study period: 3 months.	Patients with confirmed myocardial infarction N = 60	Dose: 4g per day Unstandardized capsules	Platelet aggregation—Agonist(s): ADP and Epi	Ginger had no significant effect on both measures of aggregation	India	III-1*	Ginger had no significant effect on blood lipids or blood sugar.
		Outcomes measured at: baseline, 1.5 months and 3 months.			- Method (Device, if reported): Turbidimetric				Results relating to fenugreek excluded from table.
					Fibrinogen				No mention of randomisation
					Fibrinolytic activity				P value not reported
Bordia et al. 1997	Placebo-controlled trial	Total study period: One day	Patients with confirmed myocardial infarction	Dose: 10g single dose	Platelet aggregation	Reduction of both measures of platelet aggregation when compared to placebo (p<0.05).	India	III-1	This study was detailed in same manuscript as above.
		Outcomes measured at: baseline, 4 hours post-consumption	N = 20	Unstandardized capsules	- Agonist(s): ADP and Epi				
					- Method (Device, if reported): Turbidimetric				
Janssen et al. 1996	Randomised, placebo-controlled cross-over trial	Total study period: 6 weeks (3x2 weeks)	Healthy volunteers	Dose: 15g raw & 40g cooked ginger placebo, once per day.	Thromboxane B2 production (Payton Aggregation Module)	Both types of ginger had no significant effect on maximum thromboxane B2 production (p = 0.616)	Netherlands	II	
		Outcomes measured at day 12 and 14 of each study period.	Age: 22±3	Contained within 125g custard					
			N = 18						
Jiang et al. 2004	Randomized, open label, three-way cross-over trial	Total study period: 3x13 days, 14 days washout period between each study period.	Healthy male volunteers	Dose: 3.6g (3x 0.4g, thrice per day)	Platelet aggregation	No significant changes in any outcome	Australia	III-1	No placebo group was included in study
		Outcomes measured at multiple time points, starting 2 days pre-warfarin consumption to 7 days post-consumption	Age: 20–36	Unstandardized capsules	- Agonist(s): AA				Results relating to participants receiving ginkgo supplementation were excluded from table.
			N = 12		- Method (Device, if reported): Turbidimetric (Chrono-log)				P value not reported
				Consumed with 25 mg dose of rac-warfarin, consumed once per study period.	INR				
					Plasma warfarin enantiomer protein binding & warfarin enantiomer concentrations				
					Urinary S-7-hydroxywarfarin				
Lumb. 1994	Randomised, double-blinded placebo-controlled cross-over trial	Total study period: 2x1 day, at least 14 days washout period.	Healthy male volunteers	Dose: 2g (4x500mg) dried ginger per day	Platelet aggregation	No significant changes in any outcome at any time point.	UK	II	
		Outcomes measured immediately before, 3h, and 24h post consumption of ginger	N = 8	Unstandardized capsules	- Agonist(s): AA, ADP,				
					collagen, ristocetin, ADP				
					- Method (Device, if reported): Electrical impedance (Chrono-log)				
					Bleeding time				
					Platelet count				
					Thromboelastography				
Srivastava 1989	Open-label single-arm trial	Total study period: 7 days	Health female volunteers	Dose: 5g raw ginger per day	Platelet thromboxane B2 production	Ginger consumption resulted in a 37% inhibition of thromboxane B2 production (p<0.01).	Denmark	III-3	Results relating to onion group excluded from table.
		Outcomes measured at baseline and 7 days post-consumption	N = 7						
Verma et al. 1993	Randomised placebo-controlled trial	Total study period: 14 days, high-calorie diet for first 7 days, high-calorie diet and ginger/placebo consumed for next 7 days.	Health male volunteers	Dose: 5g (4x625mg, twice per day) dry ginger powder	Platelet aggregation	Ginger significantly reduced platelet aggregation using both agonists when compared to placebo group (p<0.001).	India	II	Platelet aggregation reduced close to baseline but did not decrease further.
		Outcomes measured at baseline, 7, and 14 days	N = 20	Unstandardized capsules	- Agonist(s): ADP and Epi				
				Consumed with 100g (2x50g) butter, 2 cups of milk, 8 slices of bread.	- Method (Device, if reported): turbidimetric (ELVI-840)				
Young et al. 2006	Cross-over trial	Total study period: 72 days, 4x washout period of 7–10 days, 5x7 days intervention consumed	Healthy & Hypertensive volunteers	Dose: 1g dried ginger per day	Platelet aggregation	Ginger combined with nifedipine resulted in a significant decrease in platelet aggregation (p<0.001). Ginger alone had no significant effect.	Taiwan	III-2	No placebo group
		Outcomes measured at baseline and 7 days post-consumption for each intervention	N = 10 for each group	Either alone or in combination with 10mg nifedipine	- Agonist(s): ADP, Epi, collagen				Unclear if participants were blinded
					- Method (Device, if reported): Turbidimetric (Chronolog 560)				

Abbreviations: AA, arachidonic acid; ADP, Adenosine Diphosphate; Epi, epinephrine; INR, International Normalised Ratio; TxB_2_, Thomboxane B_2_.

* Indicates some study details were missing and that scoring was based on details available.

**Fig 2:**
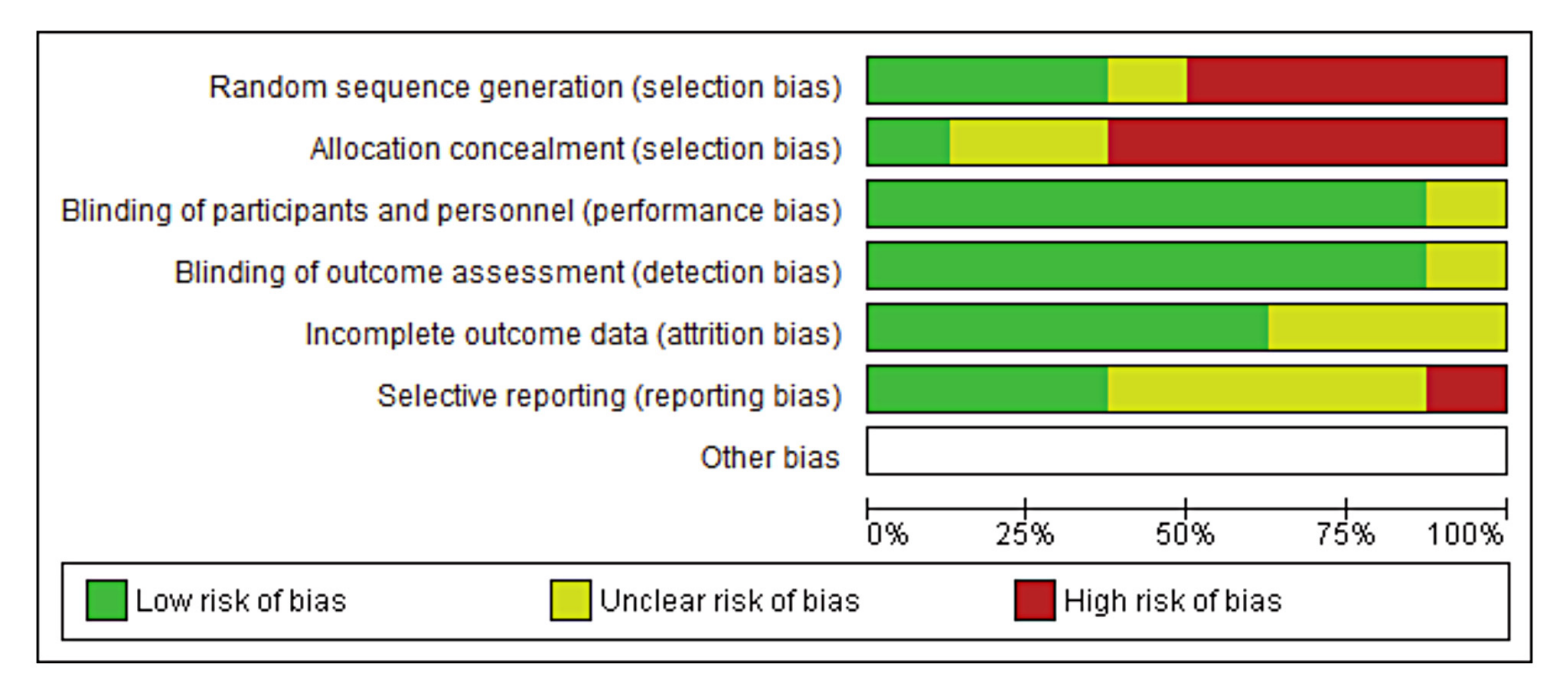
Risk of bias graph: review authors' judgements about each risk of bias item presented as percentages across all included studies.

The methodology varied considerably between trials. Half of the studies used a cross-over design[25–28] while three used a parallel design[19, 24] and one was a single arm study.[19] Most of the studies (7/8) had elements of robust study design such as placebo controls, randomisation and double-blinding. However, few studies incorporated all of these elements, with only two studies featuring both randomisation and double-blinding procedures. For example, Jiang et al.[26] used a randomised cross-over design that was also open-label (Table 1). Despite this, the assessment of bias determined the majority of studies were relatively low-risk in terms of performance, detection, and attrition bias while a high risk of random sequence generation and allocation concealment bias was detected (Fig 2).

The average sample size was small. Seven of the eight studies ranged from 7–36 participants[19, 25–29] with one study comprising 60 participants.[24] The duration of each study varied considerably, ranging from one day to three months. Six of the eight studies included healthy participants,[19, 25–27, 29] two studies included patients with confirmed myocardial infarction and one study included hypertensive patients as well as healthy participants.[24, 28] Most studies required participants to consume only ginger, either as a supplement or as a food preparation, while three studies measured the effect of ginger in combination with various medications and food products including nifedipine,[28] warfarin,[26] custard,[30] and a high-calorie diet.[29]

In terms of the ginger preparation used, seven of the eight studies tested a dose of 3.6g to 5g, while one cross-over study investigated larger doses of ginger with each participant receiving either 10g or 40g per day.[30] Most studies delivered ginger at either one time point or once per day, depending on the trial timeframe; however, Jiang et al.[26] and Verma et al.[29] delivered ginger thrice and twice per day, respectively. All studies used an unstandardized ginger preparation, either dried, cooked or raw ginger, delivered in an unprocessed form, within capsules, or mixed into a medium (i.e. custard).

Measures of platelet aggregation varied between studies. The majority (6/8) used light transmittance aggregometry or impedance aggregometry,[24, 26–29] while two studies assessed thromboxane B_2_ production.[19, 25] Three studies also recorded multiple additional outcomes including INR,[26] fibrinogen and fibrinolytic activity,[24] bleeding time, thromboelastography and platelet count.[27] Of the six that used aggregometry, there was a mix of agonists used with ADP (5/6) and epinephrine (4/6) being the most common. Three studies also used one or more of the following agonists: collagen, AA, or ristocetin.[26–28]

The reported effect of ginger on platelet function were equivocal. Two studies reported inhibition of platelet aggregation.[24, 29] The first study found that ginger significantly inhibited platelet aggregation in healthy males after consumption of a high-calorie diet.[29] The second study reported that ginger the co-administration of 1g of ginger with nifedipine resulted in an inhibition of platelet aggregation in normo- and hypertensive subjects.[28] However, in this study, when ginger was administered alone, there was no significant effect.

In contrast, two studies reported that 2–3.6g of ginger had no effect on measures of platelet aggregation in health adults.[26, 27] Moreover, Jiang et al.[26] found that the co-administration of 3.6g of ginger with 25mg of warfarin had no effect on the international normalized ratio (INR) or the pharmacokinetics and pharmacodynamics of warfarin in healthy male participants. Lumb et al.[27] also reported no significant effect on bleeding time, platelet count, and thromboelastography in a similar population. Bordia et al.[24] reported that 4g/day of ginger for three months did not affect platelet aggregation, fibrinogen, or fibrinolytic activity in patients with coronary artery disease; however, when participants were given a bolus dose of 10g ginger, there was a significant inhibition of platelet aggregation in patients with coronary artery disease.

The two studies that investigated the effect of ginger on thromboxane B_2_ generation in healthy adults reported conflicting results. Srivastava et al.[19] reported that 5g of ginger over 7 days resulted in a 37% inhibition of thromboxane B_2_ production (p<0.01), while Janssen et al.[25] found that 15g and 40g of raw and cooked ginger, respectively, had no effect when each were consumed for two weeks (p = 0.616).

### 3.2 Observational Data

Two observational studies investigated the association of ginger use and platelet-related adverse effects. Shalansky et al.[31] conducted a 16-week longitudinal study of 171 participants prescribed warfarin. During this period, participants were asked to record bleeding events as well as factors that the investigators hypothesised could influence INR and bleeding risk, including a selection of complementary therapies. Of the 171 participants, 87 reported bleeding events with excessive bruising (41%) and nosebleeds (15%) being the two most commonly-reported events. The study reported a significant association between self-reported bleeding events and ginger (OR 6.63, 95% CI 3.49–12.61), as well as cayenne (OR 8.0, 95% CI 3.57–17.92), willow bark (OR 9.00, 95% CI 6.42–12.62), St. John’s wort (OR 4.70, 95% CI 1.49–14.79), and coenzyme Q10 (OR 3.91, 95% CI 2.09–7.31). Upon further analysis, ginger (OR 3.20, 95% CI 2.42–4.24) and coenzyme Q10 (OR 3.69, 95% CI 1.88–7.24) were independently associated with self-reported bleeding events in a fully adjusted multivariate model. No complementary therapies were associated with a risk of abnormal INR.

In contrast, Leung et al.[32] surveyed 314 patients prescribed warfarin therapy, in which they retrospectively assessed self-reported bleeding events and exposure to factors that could influence bleeding risk and INR in the previous month. While only two patients reported using ginger during this period, the study authors determined that ginger, along with all other assessed complementary therapies, was not associated with bleeding risk or abnormal INR.

## Discussion

Despite consistent *in vitro* data demonstrating that ginger compounds interact with several steps involved in platelet aggregation, the results of human studies are inconsistent. It is difficult to draw conclusions from these studies as a whole, due to the limited number of studies and their heterogeneous methods. These inconsistencies include the dose, dosing regimen, and formulation of ginger used, the timeframe studied, and the characteristics of subjects recruited (e.g. healthy vs. patients with chronic diseases).

Of the eight clinical trials analysed for this review, three found ginger affected measures of platelet aggregation[24, 28, 29] and one study found ginger reduced thromboxane B_2_ production.[19] When the included studies were separated by patient medical background (e.g. healthy, hypertensive), no consistent treatment effect could be elucidated. However, there are several limitations that could limit the real-world applicability of these results.

First, Young et al.[28] reported that ginger had an effect only when it was combined with nifedipine, but not when it was ingested by itself. While not fully elucidated, it is thought that the anti-aggregation effect of nifedipine results from the inhibition of intracellular Ca2+, which attenuates platelet hyperactivity. [33] Other anti-platelet medications are not reported to possess this mechanism of action and therefore, these results might only be applicable to this combination.

Second, Verma et al.[29] found that ginger reduced a rise in platelet aggregation after a two week high-calorie diet when compared to control (high calorie diet plus placebo). However, it should be noted that this diet exceeded the participants’ normal dietary intake (approximately 1600kcal increase in dietary intake, according to USDA food data[34]), which might make these results difficult to compare to patients who consume a eucaloric diet.

The third study reported a significant reduction in platelet aggregation when a bolus of 10g ginger was administered to patients with a confirmed myocardial infarction.[24] However, the same authors found a lower dose of 4g ginger had no effect in the same population when taken daily over three months.

A primary limitation of the studies reviewed is the lack of quantification or standardisation of bioactive compounds in the ginger preparations used. This could partly explain the inconsistent results. Previous research indicates that the concentration of the principal compounds within ginger, namely gingerol and shogaols, varies greatly depending on the storage and preparation of ginger products.[35, 36] This variation could result in significant differences in bioactive compounds between studies. For example, 6-shogaol is only present in appreciable amounts in dried or heated ginger as it is a degradation product of 6-gingerol.[37] Hence, preparations that used dried ginger are likely to have significantly different effects compared to raw ginger.

A final limitation relates to the clinical significance of ginger’s potential anti-platelet effect. Several studies have reported that ginger is effective for nausea in multiple settings including morning sickness, motion sickness and chemotherapy-induced nausea and vomiting (CINV).[4, 5, 38] However, the majority of these studies used ginger doses that were considerably lower than those used in the studies included in this review. For example, in two recent reviews of the effect of ginger on morning sickness[5] and CINV[1], from a total of 19 studies, no study used a dose above 2g with most studies using a dosage around 1g. In contrast, the majority of studies in this review that found a significant effect on platelet aggregation used doses above 5g.[19, 24, 29] Young et al.[28] were the only exception in reporting 1g in combination with nifedipine to have an effect on platelet aggregation; however, when 1g of ginger was administered alone, there was no significant effect. Hence, further research in this area should investigate the effect of lower doses of ginger on platelet aggregation in order to determine if the potential effect of ginger on platelet aggregation is clinically relevant when used as an adjuvant anti-nausea treatment during chemotherapy at doses shown to be effective in previous studies.

The two observational studies included in this review also reported conflicting results.[31, 32] This could be due to the differences in their study designs. One study undertook a retrospective analysis that could have resulted in recall bias,[32] while the other study undertook a prospective approach.[31] In the retrospective study,[32] only two patients from a cohort of 314 participants reported consuming ginger, both of whom reported experiencing bleeding events. Due to the limited sample of participants who consumed ginger, it is difficult to draw meaningful conclusions. Information regarding the dose of ginger consumed by participants was not reported in either observational study, which might further account for the difference in results.

While there was only one clinical trial investigating the interaction between ginger and warfarin, Jiang et al.[26] found no significant change to patient INR when ginger was administered for seven days. This is partially corroborated by the results of a study of Wistar rats in which a proprietary ginger formulation, in combination with warfarin, had no additive effect on whole blood clotting time, prothrombin time or activated partial thromboplastin time.[10] This is a particularly relevant finding, as ginger is routinely cited as potentially interacting with warfarin therapy.[39, 40] While further studies are required to investigate interaction of ginger and blood thinning medication, current evidence does not support an interaction.

The results of this review indicate that the role of ginger in platelet aggregation is unclear and therefore, future clinical trials are needed to further investigate this area, particularly in at-risk populations such as chemotherapy patients. However, until these trials are undertaken, the effect of ginger on platelet aggregation cannot be confidently dismissed. Previous research has indicated that patient use of dietary supplements is often not reported to treating physicians. For example, a review of surveys that investigated the rate of non-disclosure of complementary and alternative medicines in chemotherapy patients found that between 40–50% of patients did not discuss these therapies with their physician.[41] Hence, where patients are at particular risk of bleeding, clinicians should ascertain patient consumption of dietary supplements and screen for any known potentiator of bleeding risk.

## Conclusion

Due to the potential effects of ginger on platelet aggregation, ginger is a commonly-cited example of an herbal supplement that should be avoided in patients with thrombocytopenia, platelet function defects or coagulopathy, such as populations using ginger for its antiemetic effect in cancer chemotherapy. While *in vitro* data, as well as some clinical studies and epidemiological evidence suggest that ginger inhibits platelet aggregation, the evidence is equivocal with multiple limitations, particularly within the clinical data, which prevents firm recommendations being made. Limitations include the lack of standardisation of ginger preparations used, significant variations in dosage and time frame studied, and the high level of bias in the study designs used. Therefore, further research is needed to clearly define the safety, or otherwise, of ginger in patient population at increased risk of bleeding.

## Supporting Information

S1 File PRISMA Guidelines Checklist. (DOC)
