# Correction: The Effect of Ginger (*Zingiber officinale*) on Platelet Aggregation: A Systematic Literature Review

**DOI:** 10.1371/journal.pone.0143675

**Published:** 2015-11-23

**Authors:** Wolfgang Marx, Daniel McKavanagh, Alexandra L. McCarthy, Robert Bird, Karin Ried, Alexandre Chan, Liz Isenring

Table 1 appears incorrectly in the published article. Please see the corrected Table 1 here.

**Table 1:** Extraction table of reviewed clinical trials.

Author/Date	Bordia et al. 1997	Bordia et al. 1997	Janssen et al. 1996	Jiang et al. 2004	Lumb. 1994	Srivastava 1989	Verma et al. 1993	Young et al. 2006
Study design	Placebo-controlled trial	Placebo-controlled trial	Randomised, placebo-controlled cross-over trial	Randomized, open label, three-way cross-over trial	Randomised, double-blinded placebo-controlled cross-over trial	Open-label single-arm trial	Randomised placebo-controlled trial	Cross-over trial
Sample Size	N = 60	N = 20	N = 18	N = 12	N = 8	N = 7	N = 20	N = 10 for each group
Total study period:	3 months.	One day	6 weeks (3x2 weeks)	3x13 days, 14 days washout period between each study period.	2x1 day, at least 14 days washout period.	7 days	14 days, high-calorie diet for first 7 days, high-calorie diet and ginger/placebo consumed for next 7 days.	72 days, 4x washout period of 7–10 days, 5x7 days intervention consumed
Population	Patients with confirmed myocardial infarction	Patients with confirmed myocardial infarction	Healthy volunteers	Healthy male volunteers	Healthy male volunteers	Health female volunteers	Health male volunteers	Healthy & Hypertensive volunteers
Outcomes measured at:	Baseline, 1.5 months and 3 months.	Outcomes measured at: baseline, 4 hours post-consumption	Outcomes measured at day 12 and 14 of each study period.	Outcomes measured at multiple time points, starting 2 days pre-warfarin consumption to 7 days post-consumption	Outcomes measured immediately before, 3h, and 24h post consumption of ginger	Outcomes measured at baseline and 7 days post-consumption	Outcomes measured at baseline, 7, and 14 days	Outcomes measured at baseline and 7 days post-consumption for each intervention
Intervention	Dose: 4g per day of unstandardized capsules	Dose: 10g single dose of unstandardized capsules	Dose: 15g raw & 40g cooked ginger placebo, once per day. Contained within 125g custard	Dose: 3.6g (3x0.4g, thrice per day) of unstandardized capsules. Consumed with 25 mg dose of rac-warfarin, consumed once per study period.	Dose: 2g (4x500mg) dried ginger per day of unstandardized capsules	Dose: 5g raw ginger per day	Dose: 5g (4x625mg, twice per day) dry ginger powder of Unstandardized capsules. Consumed with 100g (2x50g) butter, 2 cups of milk, 8 slices of bread.	Dose: 1g dried ginger per day, either alone or in combination with 10mg nifedipine
Outcome	Platelet aggregation	Platelet aggregation	Thromboxane B2 production (Payton Aggregation Module)	Platelet aggregation	Platelet aggregation	Platelet thromboxane B2 production	Platelet aggregation	Platelet aggregation
	- Agonist(s): ADP and Epi	- Agonist(s): ADP and Epi		- Agonist(s): AA	- Agonist(s): AA, ADP, collagen, ristocetin		- Agonist(s): ADP and Epi	- Agonist(s): ADP, Epi, collagen
	- Method (Device, if reported): Turbidimetric	- Method (Device, if reported): Turbidimetric		- Method (Device, if reported): Turbidimetric (Chrono-log)	- Method (Device, if reported): Electrical impedance (Chrono-log)		- Method (Device, if reported): turbidimetric (ELVI-840)	- Method (Device, if reported): Turbidimetric (Chronolog 560)
	Fibrinogen			INR	Bleeding time			
	Fibrinolytic activity			Plasma warfarin enantiomer protein binding & warfarin enantiomer concentrations	Platelet count			
				Urinary S-7-hydroxywarfarin	Thromboelastography			
Results	Ginger had no significant effect on both measures of aggregation	Reduction of both measures of platelet aggregation when compared to placebo (p<0.05).	Both types of ginger had no significant effect on maximum thromboxane B2 production (p = 0.616)	No significant changes in any outcome	No significant changes in any outcome at any time point.	Ginger consumption resulted in a 37% inhibition of thromboxane B2 production (p<0.01).	Ginger significantly reduced platelet aggregation using both agonists when compared to placebo group (p<0.001).	Ginger combined with nifedipine resulted in a significant decrease in platelet aggregation (p<0.001). Ginger alone had no significant effect.
Country	India	India	Netherlands	Australia	UK	Denmark	India	Taiwan
Level of evidence	III-1*	III-1	II	III-1	II	III-3	II	III-2
Comment	Ginger had no significant effect on blood lipids or blood sugar. Results relating to fenugreek excluded from table. No mention of randomisation. P value not reported.	This study was detailed in same manuscript as previous study.		No placebo group was included in study. Results relating to ginkgo group excluded from table. P value not reported.		Results relating to onion group excluded from table.	Platelet aggregation reduced close to baseline but did not decrease further.	No placebo group. Unclear if participants were blinded.

Abbreviations: AA, arachidonic acid; ADP, Adenosine Diphosphate; Epi, epinephrine; INR, International Normalised Ratio; TxB2, Thromboxane B2;.

* Indicates some study details were missing and that scoring was based on details available.
